# Medical Experts as Witnesses in Courts of Justice

**Published:** 1878-05

**Authors:** F. T. Lederberger

**Affiliations:** St. Louis.


### MISCELLANEOUS.

### Medical Experts as Witnesses in Courts of Justice.

BY F. T. LEDERBERGER, ESQ., of St. Louis.

Scientists or experts can only give an opinion as to the probable effect of certain acts or circumstances, hypothetically stated. But they are not permitted to give an opinion on the point of inquiry, which the jury must decide. This would be in effect, to decide the case for them, as it is termed in law, taking the case from the jury, an infringement upon its special province, and of the right of parties to have it tried by the jury. The sole object of this testimony is to throw light upon the subject matter of the inquiry at issue in the case on trial. It would defeat the object sought to be accomplished, if the witness brought into Court for the purpose of aiding the jury in coming to the right conclusion, were permitted by his opinion, to say that which ought and can only be determined by the jury. Therefore scientists or experts cannot, by their opinion, determine or give their opinion upon the point the jury is to decide.

It is therefore improper to ask a witness what in his opinion would be the effect, but the question should conclude, what might be the effect, or what would be the effect, or what would be the probable effect or result. To illustrate, in a trial for the larceny of a certain article of value, the attending physician cannot testify that in his opinion the taking was an act of insanity. He can only state that from all the facts it is his opinion that "the defendant was probably laboring or may have been laboring under a fit of insanity at or about the time of the taking." It is true, if the jury believe him, the inference is the same, but it alone can find the fact, and then act upon it. The jury may still find that the person was sane at the taking.

Scientists and experts are the only witnesses who are permitted or compelled to testify in a case, the facts of which are unknown to them, and which they have not heard detailed by the other witnesses. Hypothetical cases are put to them for their opinion as to the probable result or probable effect.[1] Where such a witness gives an opinion upon facts witnessed by himself, he must state the facts upon which he basis it. The reason for the above is evident, for if he could give his opinion without divulging the facts upon which it was founded, it would prevent others from understanding him, and what is of more importance, make him the judge and not merely a witness in the case. It would withhold evidence from the jury, upon which they must pass.[2] The facts upon which an opinion is asked must be undisputed, and not put in the alternative,[3] for opinious upon different states of facts would not assist either the Court or jury in arriving at a correct understanding, but the contrary. This does not exclude counsel on either side from asking an opinion upon a state of facts they suppose to exist or have tried to prove in the case. The supposed case must be clear and relevant to the evidence in the case, leaving it to the jury to find the facts and then apply the opinion. No opinion can be

---

1. U. S. vs. McGlue, 1st Curtis, 1; Perkins vs. Concord, 44 N. H., 223, 1 Parker, N. Y., 495, 48 Mo., 291; State vs. Smith, 32 Me., 369; Lake vs. People, 12 N. Y., 358; Stephens vs. People, 4 Parker (N. Y.,) p 396.

2. 26 Wend, People vs. Lake, 32 N. Y., 358, 8 Mass., 371, Gib. 225; White vs. Bailey, 10 Mich., 155.

3. U. S. vs. McGlue, *supra*.

given on a part of the facts in the case.[4] The soundness of this rule no one will doubt, the object being to enlighten and not to confuse the triers of facts.

An experienced physician, after having made a post mortem examination of the body of a female, may as an expert, offer his opinion as to whether she had been pregnant and what was the probable cause of her death.[5] Whether certain symptoms, particularly specified, were those of arsenical poisoning, the symptoms being the same related by another witness at the trial;[6] as to the nature of the affection complained of, its causes and curability;[7] as to the condition of a patient they have seen;[8] as to the kind and nature of the instrument causing the wound; in a murder case whether the wounds or injuries were likely to be the cause of death; whether certain circumstances would likely produce a paroxysm of the disorder. A physician cannot give his opinion as to whether a rape could be committed in a certain way or position, or under peculiar circumstances, nor as to the propable position of the body of the deceased when struck;[9] nor as to whether a fracture of a skull produced in Court is from a weapon also produced.[10]

The three last cases in connection with the cases where such testimony was admitted, and what has been said will tend to illustrate the rule, by which some cases are classified as proper for an opinion, and others not. That which is evident or comprehensible to persons of ordinary understanding cannot be the subject of an opinion for a scientist or expert.

There is only one more point in connection with the testimony of this class of witnesses necessary to be mentioned. This question seems to be misunderstood by many. It is as to the weight and conclusiveness of the opinions of scientists and experts. In France and Germany this has met the eye of the law makers, and received a solution, certainly more satisfactory to Court and counsel, than under the English laws relating to evidence and witnesses. There, this class of witnesses are called on by the Court to investigate the subject matter of inquiry in the case upon which their testimony is required. They are clothed with the authority of the Court during their investigation and report to the Court, and unless bias, fraud, or partiality be shown, the Court adopts their opinion as the rule of law in that case. The Courts awards them suitable compensation. They cannot well become partisans for one side, for neither side calls them. True it is, that not so many can attain

---

4. People vs. Lake, 12 N. Y., 358 Herald vs. Thing, 45 Maine, 392.
5. State vs. Smith, *supra*.
6. Stephens vs. People, *supra*.
7. Matteson vs. N. Y. C., 35 N. Y., 487.
8. State vs. Smith, 32 Me., 369. Lake vs. People, 12 N. Y., 358.
9. Kennedy vs. People, 39 N. Y., 245.
10. Wilson vs. People, 4 Parker N. Y., 619.

the prominence of testifying in a case, and that only men standing high in their profession or art are appointed.

Under our system it frequently happens that quite a number of scientists or experts are subpœned on each side, and strange as it may seem, in most instances a majority give their opinion favorable to the party calling them, and it seldom, if ever occurs, that one of them will say that he is not thoroughly prepared or competent to give an opinion on the question propounded. The tendency of this is, to detract from the value of such opinions in the eyes of the juries and the bar. "Who shall decide, when doctors disagree?"

Opinions of scientists are to be received and weighed by the jury, and their creditability considered in the same manner as other evidence, and it is error for the Court to instruct the jury to give any more weight or evidence to their opinions. Taken in connection with the case of The People vs. Harking, *supra*, in which an expert was allowed to shake the foundation of a physician's opinion, by giving his opinion that his science was uncertain, the position scientists and experts occupy in our Courts are less satisfactory in regard to the value and effect of their testimony as such, than in regard to their pay. Should any movement be made at all, it might comprise both, so that fair pay be at least united with a position in Court worthy of the same.—*St. Louis Med. and Surg. Journal.*

